# A Novel STK4 Mutation Impairs T Cell Immunity Through Dysregulation of Cytokine-Induced Adhesion and Chemotaxis Genes

**DOI:** 10.1007/s10875-021-01115-2

**Published:** 2021-08-24

**Authors:** Andrea Guennoun, Salim Bougarn, Taushif Khan, Rafah Mackeh, Mahbuba Rahman, Fatima Al-Ali, Manar Ata, Waleed Aamer, Debra Prosser, Tanwir Habib, Evonne Chin-Smith, Khawla Al-Darwish, Qian Zhang, Alya Al-Shakaki, Amal Robay, Ronald G. Crystal, Khalid Fakhro, Amal Al-Naimi, Eman Al Maslamani, Amjad Tuffaha, Ibrahim Janahi, Mohammad Janahi, Donald R. Love, Mohammed Yousuf Karim, Bernice Lo, Amel Hassan, Mehdi Adeli, Nico Marr

**Affiliations:** 1 Research Branch, Sidra Medicine, PO BOX 26999, Doha, Qatar; 2 Present Address: Translational Cancer and Immunity Center, Qatar Biomedical Research Institute, Doha, Qatar; 3 Department of Pathology, Sidra Medicine, Doha, Qatar; 4 Present Address: Bioinformatics Core, Weill Cornell Medicine-Qatar, Doha, Qatar; 5 St. Giles Laboratory of Human Genetics of Infectious Diseases, Rockefeller Branch, The Rockefeller University, New York, NY USA; 6 Weill Cornell Medicine-Qatar, Doha, Qatar; 7 Weill Cornell Medicine, New York, NY USA; 8 College of Health and Life Sciences, Hamad Bin Khalifa University, Doha, Qatar; 9 Department of Pediatrics, Sidra Medicine, Doha, Qatar

**Keywords:** Human serine/threonine kinase 4 (STK4) deficiency, Combined immunodeficiency, T cell lymphopenia, Interferon, Antibody repertoire, Transcriptomics

## Abstract

**Purpose:**

Human serine/threonine kinase 4 (STK4) deficiency is a rare, autosomal recessive genetic disorder leading to combined immunodeficiency; however, the extent to which immune signaling and host defense are impaired is unclear. We assessed the functional consequences of a novel, homozygous nonsense STK4 mutation (NM_006282.2:c.871C > T, p.Arg291*) identified in a pediatric patient by comparing his innate and adaptive cell-mediated and humoral immune responses with those of three heterozygous relatives and unrelated controls.

**Methods:**

The genetic etiology was verified by whole genome and Sanger sequencing. STK4 gene and protein expression was measured by quantitative RT-PCR and immunoblotting, respectively. Cellular abnormalities were assessed by high-throughput RT-RCR, RNA-Seq, ELISA, and flow cytometry. Antibody responses were assessed by ELISA and phage immunoprecipitation-sequencing.

**Results:**

The patient exhibited partial loss of STK4 expression and complete loss of STK4 function combined with recurrent viral and bacterial infections, notably persistent Epstein–Barr virus viremia and pulmonary tuberculosis. Cellular and molecular analyses revealed abnormal fractions of T cell subsets, plasmacytoid dendritic cells, and NK cells. The transcriptional responses of the patient’s whole blood and PBMC samples indicated dysregulated interferon signaling, impaired T cell immunity, and increased T cell apoptosis as well as impaired regulation of cytokine-induced adhesion and leukocyte chemotaxis genes. Nonetheless, the patient had detectable vaccine-specific antibodies and IgG responses to various pathogens, consistent with a normal CD19 + B cell fraction, albeit with a distinctive antibody repertoire, largely driven by herpes virus antigens.

**Conclusion:**

Patients with STK4 deficiency can exhibit broad impairment of immune function extending beyond lymphoid cells.

**Supplementary Information:**

The online version contains supplementary material available at 10.1007/s10875-021-01115-2.

## Introduction

Human serine/threonine kinase 4 (STK4) deficiency is a rare autosomal recessive (AR) genetic disorder leading to combined immunodeficiency with severe T cell lymphopenia. This condition is characterized by a predisposition to a wide range of bacterial and viral infectious diseases, mucocutaneous candidiasis, lymphomas, and congenital heart disease [1]. To date, STK4 deficiency has been reported in relatively few patients; therefore, the extent to which immune signaling and host defense mechanisms are impaired or dysregulated in affected individuals remains incompletely understood. However, the spectrum of clinical manifestations associated with STK4 deficiency has been steadily increasing with each new case report.

STK4 deficiency was first reported by Nehme et al. in two patients from unrelated Turkish families harboring a homozygous nonsense mutation in the *STK4* gene [2]. The patients experienced complications due to recurrent bacterial and viral infections, most notably persistent Epstein–Barr virus (EBV) viremia, which ultimately resulted in Hodgkin B cell lymphoma. Due to weak expression of the homing receptors CCR7 and CD62L, the authors attributed the underlying mechanism of STK4 deficiency to increased death of naïve and proliferating T cells, and impaired homing of CD8^+^ T cells to secondary lymphoid organs [2]. Abdollahpour et al. reported the cases of three siblings of Iranian descent with a homozygous premature stop codon in the *STK4* gene [3]. These patients suffered from T and B cell lymphopenia, intermittent neutropenia, and atrial septal defects, as well as recurrent bacterial and viral infections, mucocutaneous candidiasis, cutaneous warts, and skin abscesses. Interestingly, Schipp et al. reported a Turkish patient with STK4 deficiency who developed a highly malignant B cell lymphoma at the age of 10 years and a second, independent Hodgkin lymphoma 5 years later. However, no detectable EBV or other common virus infection was detected in this patient. The authors speculated that the lymphoma may have developed due to the lack of the tumor suppressive function of STK4 or perturbed immune surveillance due to the diminished CD4^+^ T cell compartment [4]. In contrast, most malignancies reported in patients with STK4 deficiency are associated with prolonged EBV viremia, ultimately leading to the development of B cell lymphomas [2, 5–7]. More specifically, patients present with Hodgkin B cell lymphoma [2], extranodal marginal zone lymphoma of mucosa-associated lymphoid tissue [8], Burkitt’s lymphoma [7], or maxillary sinus diffuse large B cell lymphoma [9]. Additional clinical features in patients with STK4 deficiency include salt-losing tubulopathy, suggestive of an acquired Gitelman syndrome, immune complex glomerulonephritis, and Castleman-like disease [10], juvenile idiopathic arthritis [11], human beta-papillomavirus-associated epidermodysplasia verruciformis [11, 12], primary cardiac T cell lymphoma [6], and short stature [13].

Studies in mice and humans have shown that STK4 plays a pivotal role in lymphocyte function by regulating integrin-dependent T lymphocyte trafficking, proliferation, and differentiation [14, 15]. Of note, the STK4 protein is broadly expressed in various human hemopoietic cells, most notably monocytes, and is not restricted to lymphocytes (https://www.proteinatlas.org/ENSG00000101109-STK4). However, its role in T cell-independent functions is less well understood. Recently, Jørgensen et al. studied innate immune signaling in the context of STK4 deficiency by in vitro stimulation or infection of PBMCs obtained from an 11-year-old female STK4^−/−^ patient of a consanguineous Syrian family. These studies revealed defective type I/II and III interferon (IFN) responses to a variety of purified Toll-like receptor (TLR) agonists, live viruses and bacterial lysates due to impaired phosphorylation of the kinase TBK1 and the transcription factor IRF3 [13]. The results also revealed increased apoptosis in STK4-deficient T cells and neutrophil granulocytes, possibly linked to defective FOXO signaling in STK4-deficient T cells as shown in earlier studies [2, 3], further supporting the important role of STK4 in T cell survival.

In this study, we identified an AR STK4 deficiency in a child from consanguineous parents, which was due to a novel homozygous stop-gain mutation in a region encoding a coiled-coil domain located downstream of the kinase domain. We investigated the functional consequences of the new variant on innate and adaptive cell-mediated and humoral immune responses.

## Results

### Clinical Description of the Case and Family Members

**Fig. 1:**
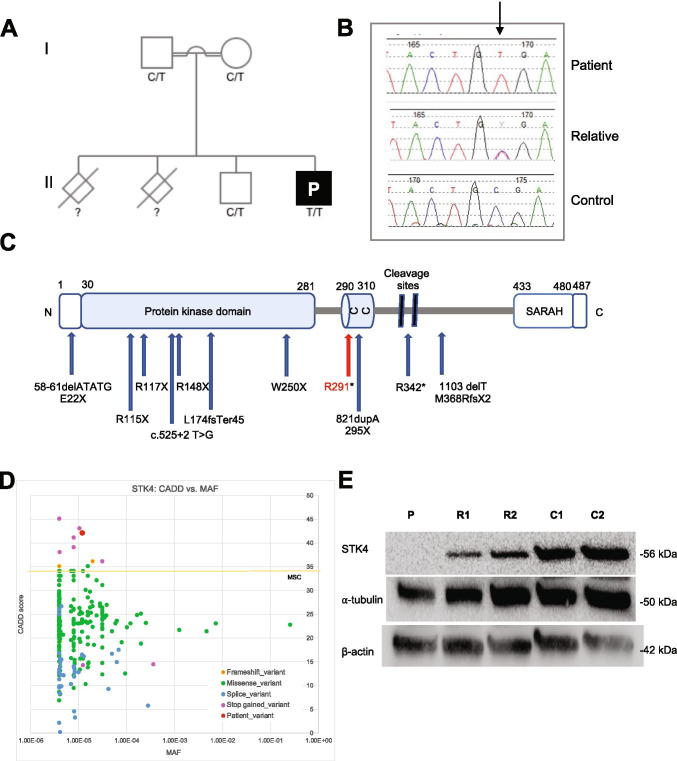
Identification of a homozygous *STK4* gene mutation in a patient from consanguineous parents. **A** Pedigree and segregation of the *STK4* gene mutation. The patient (P) is homozygous for the mutation. Question marks (?) indicate individuals whose genetic status could not be evaluated. **B** Electropherograms of partial sequences of *STK4* corresponding to the mutation in a healthy control (bottom), patient (up), and a STK4^wt/mut^ relative (middle) representative of all three healthy family members. The reference vs. altered nucleotide position is indicated by a black arrow. **C** Schematic illustration of the protein encoded by the *STK4* gene, with domain boundaries and other features retrieved from the UniProtKB (www.uniprot.org) (entry Q13043). Blue arrows indicate previously reported variants [2–5, 9–13, 20]. The variant identified in P is indicated in red. CC, coiled coil domain; SARAH, Sav/Rassf/Hpo domain (IPR024205). **D** Data from the gnomAD database were used to plot minor allele frequency (MAF) against the Combined annotation-dependent depletion (CADD) score values of all known variants in *STK4* and the variant identified in P. **E** Western blot analysis of STK4 protein expression in PBMC-derived T lymphocytes from P, two STK4^wt/mut^ heterozygous relatives (R1 and R2) and two unrelated STK4^wt/wt^, healthy controls (C1 and C2); a-tubulin and b-actin antibodies were used as controls

The patient was the third child (male) of consanguineous parents (first-degree cousins). The patient had a younger sibling who was healthy, as were both parents. Of note, the parents reported the death of the patient’s two elder siblings; one died with a history of chronic headache, coughing, and lymphoma, while the other died with a history of chronic coughing (Fig. 1A). However, no detailed medical records or genetic data were available for the two deceased siblings.

The patient suffered from recurrent skin rashes starting from infancy, recurrent chest infections since early childhood, and an overall failure to thrive with low weight gain and short stature (data not shown), consistent with previous reports of other patients with STK4 deficiency [13]. The patient’s early medical history included a productive cough of yellow/white mucoid sputum associated with intermittent fever, which was more common at bedtime; however, early medical records were very limited and likely to be incomplete. After closer clinical monitoring starting at elementary school age, the patient was diagnosed with bronchiectasis and asthma, and started on asthma control medication, including Ventolin. He also experienced complications due to recurrent viral and bacterial infections, chronic suppurative otitis media, and recurrent pneumonia. The patient was also diagnosed with tuberculosis (TB), which was confirmed by *Mycobacteria tuberculosis* complex-positive culture, while the result of a QuantiFERON assay performed in parallel was “indeterminate.” The patient was treated for pulmonary TB for approximately 1 year. However, a year after stopping treatment, the patient suffered from TB reactivation and was put on anti-TB medication (cycloserine, linezolid, moxifloxacin, and pyrazinamide) for a further 2 years. As a teenager, he also presented with a lower chest infection, and a chest X-ray confirmed lower left consolidation. A sputum culture revealed abundant growth of *Haemophilus influenzae* and *Streptococcus pneumoniae*, as well as modest growth of methicillin-resistant *Staphylococcus aureus*. About a year later, the patient was hospitalized with a second episode of lower chest infection by *H. influenzae* and multiple-drug-resistant *Klebsiella pneumoniae*. EBV viremia was also detected during the early teenage years and persisted to the most recent follow-up (Supplementary Table S1). During his teenage years, the patient also suffered from intermittent neutropenia and severe lymphopenia (Supplementary Table S2) with markedly decreased naïve CD45RA^+^ cells (11.1%; normal range 46–77%); the onset may have been earlier but was not detected due to the late diagnosis. The patient had received BCG vaccination at birth, as well as OPV, MMR, varicella, and meningococcal vaccines at school age. Antibody responses to childhood vaccination were within the normal range (Supplementary Table S3).

### Homozygosity for a Stop-Gain Mutation in the STK4 Gene

Whole genome sequencing revealed a rare, homozygous nonsense mutation in the *STK4* gene (NM_006282.2:c.871C > T, p.Arg291*) in the patient, whereas both parents and the younger sibling were identified as heterozygous carriers, suggesting an AR inheritance pattern (Fig. 1A). The *STK4* genotypes of the patient and his family members were confirmed by clinical Sanger sequencing (Fig. 1B). The combined annotation-dependent depletion (CADD) score of the variant was 42, providing further evidence of its deleteriousness (Fig. 1C and D).

### The mutant STK4 Allele is a LOF Variant

The mutant STK4 protein was not detected in PBMC-derived T cells from the patient by Western blot analysis using a monoclonal antibody directed against the *N*-terminus of the protein, whereas intermediate STK4 protein levels were detected in the parents compared to two unrelated healthy controls (Fig. 1E). A *STK4* transcript was detected by mRNA-Seq and RT-qPCR in PBMC-derived T cells of the patient, albeit at reduced levels compared to controls with a wild-type genotype and the heterozygous parents (Supplementary Figure S1A and B).

### Reduced Fractions of naïve T Helper, and Dendritic Cell Subsets, as well as Increased Effector Memory and Apoptotic T Helper and Precursor NK Cells in the PBMCs of the Patient

**Fig. 2:**
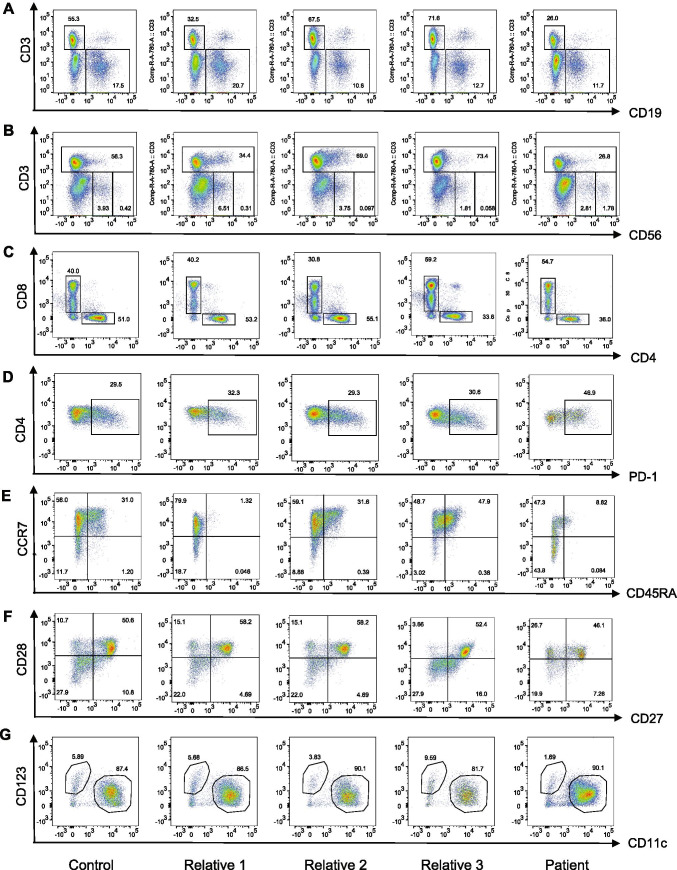
Leukocyte subsets in the STK4-deficient patient, his parents and sibling, and one unrelated healthy control. For all experiments, subjects are presented in the following order from left to right: Unrelated control, the patient’s three relatives, and the patient (P). **A** Frequency of B (CD3^−^CD19^+^) and T (CD3^+^CD19^−^) lymphocytes among CD45^+^ lymphocytes. **B** Frequency of T lymphocytes (CD3^+^) and NK cell immunophenotyping, showing the frequency of CD56^bright^ (CD3^+^CD56^bright^) and CD56^dim^ (CD3^+^CD56^dim^) NK cells among CD45^+^ lymphocytes. **C** Frequency of cytotoxic (CD3^+^CD8^+^) and helper (CD3^+^CD4^+^) T lymphocytes among the CD3^+^ lymphocyte subset. **D** Frequency of PD-1^+^ T lymphocytes (CD4^+^PD-1^+^) among the CD4^+^ T cell subset. **E** Frequency of naïve (CD45RA^+^CCR7^+^), central memory (CD45RA^−^CCR7^+^), effector memory (CD45RA^−^CCR7^−^) and effector memory cells re-expressing CD45RA (T_EMRA_) (CD45RA^+^CCR7^−^) cells among the CD4^+^ T cell compartment. **F** Frequency of CD27^+^ and CD28^+^ T helper subsets within the CD4^+^ compartment. **G** Frequency of myeloid dendritic cells (mDCs) (CD123^−^CD11c^+^) and plasmacytoid dendritic cells (pDCs) (CD123^+^CD11c^−^) among the CD45^+^HLA-DR^+^CD3^−^CD14^−^CD19^−^, CD20^−^CD56^−^ dendritic cell population

We performed polychromatic flow cytometric analyses of PBMCs obtained from the patient at middle-school-age to compare the lymphocyte subset distribution with that of his parents, his younger sibling, and one unrelated control (Fig. 2). As expected, we found a lower fraction of T cells in the patient compared to the controls (Fig. 2A, B and C), which was mainly attributed to selective CD4^+^ T cell lymphopenia (Fig. 2C). The CD19^+^ B cell population was not affected in the patient (11.7% vs. control range 10.9–20.8%) (Fig. 2A). Similarly, the patient’s CD56^+^CD3^−^ NK cells were within the normal range (4.59% vs. control range 1.87–6.82%) (Fig. 2B). However, we noticed a significant increase in the CD56^bright^ NK cell subset in the patient (1.78% vs. control range 0.058–0.42%) (Fig. 2B). We then assessed CD4^+^ T cell subsets and found an increase in programmed death-1 (PD-1)-expressing T helper cells in the patient (Fig. 2D). Further analysis of PBMC expression of CD45RA and CCR7 revealed low frequencies of CD45RA^+^CCR7^+^ double-positive naïve T cells in the patient, while his CD45RA^−^CCR7^−^ effector memory population was increased (Fig. 2E). Similarly, the CD27^+^CD28^+^ T cell subset, which consists mainly of naïve T cells, was also slightly decreased in the patient (Fig. 2F). Finally, assessment of the dendritic cell (DC) subsets showed a decrease in the CD11c^−^CD123^+^ plasmacytoid DC (pDC) population in the patient, while his CD11c^+^CD123^−^ myeloid DCs (mDCs) population remained normal (Fig. 2G).

### The Patient has a Distinct Antiviral Antibody Repertoire

**Fig. 3:**
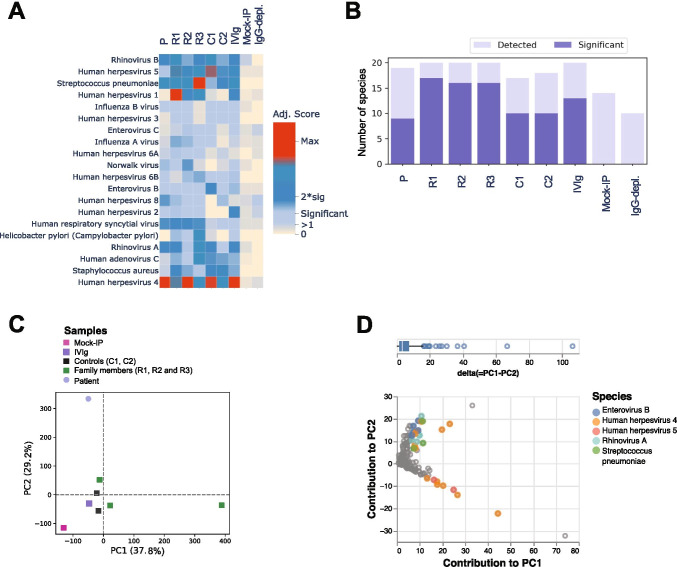
Microbial exposure profile and antiviral antibody repertoire in the STK4-deficient patient. **A** Antibody profile in the STK4^−/−^ patient (P), his STK4^WT/−^ family members (R1, R2, and R3), and two unrelated STK4^WT/WT^ controls (C1 and C2). Pooled human plasma was used for intravenous immunoglobulin therapy (IVIg); human IgG-depleted serum (IgG-depl.) and mock-IP samples served as additional controls. All samples were assayed in duplicate, and the results are derived from one experiment. Heatmap shows species-specific adjusted score values, which served as a quantitative measure of the number of antibody specificities targeting a given microbial species. **B** Bar plot depicting, for each sample shown in **A**, the number of species for which peptides were significantly enriched by PhIP-Seq (i.e., at least one antibody specificity was detected) (light blue) and the number of species for which the adjusted virus score values passed the significance cut-off (i.e., the sample was considered seropositive for that given species) (dark blue). **C** Principal component (PC) analysis of the -log_10_(*P*-values) of significantly enriched peptides for each sample as shown in **A**. **D** Scatter plot showing the contribution of the significantly enriched peptides to PC1 and PC2 in the patient’s sample. Peptides depicted in color correspond to species for which more than two peptides were enriched and had a delta (PC1-PC2) in excess of 70th percentile (top)

To further assess the humoral immunity status of the patient, we performed large-scale serum antibody profiling by phage immunoprecipitation-sequencing (PhIP-Seq). The patient was seropositive for antibodies specific to a variety of common viruses and bacteria, including human herpesviruses (HHV)-4 (EBV), -5 (CMV) and -8; enterovirus (EV)-B; and human respiratory syncytial virus, human rhinoviruses A and B, *S. pneumoniae* and *S. aureus* (Fig. 3A). The antibody repertoire breadth in the patient was similar to that of the controls (Fig. 3B). Nevertheless, PCA of the enriched antibody-antigen interactions showed an overall distinct antibody repertoire in the patient compared to those of his family members and unrelated controls (Fig. 3C). These differences were largely driven by antibodies directed against structural proteins of human herpesviruses (HHV)-4 and -5, which was consistent with the patient’s active EBV viremia (Fig. 3D).

### Gene Expression Signatures Suggest Dysregulated Interferon Signaling and Impaired T Cell Activation, Inhibition of T Cell Proliferation, and Increased T Cell Apoptosis

**Fig. 4:**
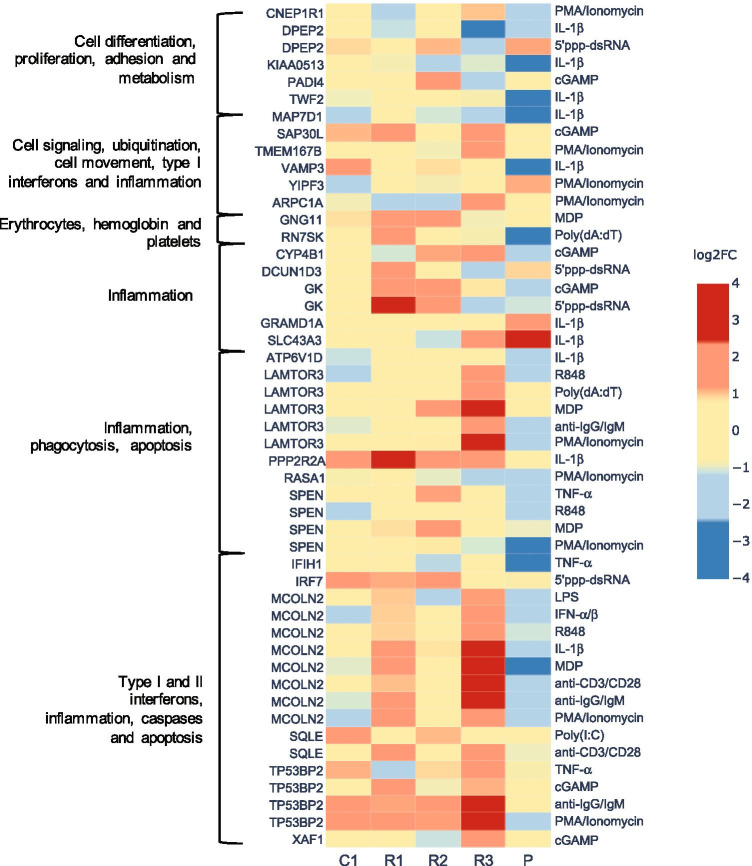
Unique gene expression signature in whole blood samples from the STK4-deficient patient following in vitro stimulation. Heatmap showing the log_2_-transformed fold change values (log2FC) of the differentially expressed genes (DEGs) among the 180 target genes for which transcriptional responses of the patient’s (P) whole blood samples to in vitro stimulation showed a variance of |log2FC|> 1 compared to those of the other family members (R1, R2, and R3) and an unrelated control (C1). Gene-stimuli pairs are grouped according to the functional annotation of the gene cluster as described previously [16]. Results were obtained from one experiment. The target genes represented 60 functionally annotated transcriptional modules (i.e., sets of co-expressed genes), with each module represented by three target genes. Full gene names and functional annotation are detailed in Supplementary Tables S3 and S4

To further elucidate the functional consequences of STK4 deficiency at the molecular level, we performed gene expression analyses using either whole blood (WB) samples or PBMCs isolated from the patient and control subjects following in vitro stimulation with a variety of immune activators. First, we stimulated WB of the patient, his family members, and an unrelated control subject with purified pattern recognition receptor (PRR) agonists (lipopolysaccharides of *Escherichia coli* K12 (LPS_K12_) (TLR4 agonist), muramyl dipeptide (MDP) (NOD2 agonist), Poly(I:C) (TLR3 agonist), Poly(dA:dT) (multiple-PRR agonist), resiquimod (R848) (TLR7/8 agonist), cyclic guanosine monophosphate-adenosine monophosphate (cGAMP) (STING agonist), and 5′ triphosphate double-stranded RNA (5'ppp-dsRNA) (RIG-I agonist)), cytokines (IFN-a/IFN-b (an interferon-α/β receptor IFNAR agonist), IFN-g, IL-1b, and TNF-a), a potent mitogen (phorbol 12-myristate 13-acetate (PMA)/ionomycin), and BCR or TCR activators. We measured the expression of 180 functionally well-annotated target genes selected a priori from a larger set of genes responsive to WB stimulation with purified PRR agonists, recombinant cytokines, and pyogenic bacteria [16] (see “Methods” and Supplementary Table S5, Online Resource 1); mock stimulations served as controls. Among these target genes, we then filtered for differentially expressed genes (DEGs) for which the transcriptional responses of the patient’s WB samples to any of the in vitro stimulation conditions showed high variance compared with those of the other family members and the unrelated control (Supplementary Figure S2). The identified DEGs were associated with caspases and apoptosis, type I and II interferon signaling, inflammation, cell signaling, and ubiquitination, as well as cell movement and phagocytosis (Fig. 4 and Supplementary Table S6).

**Fig. 5:**
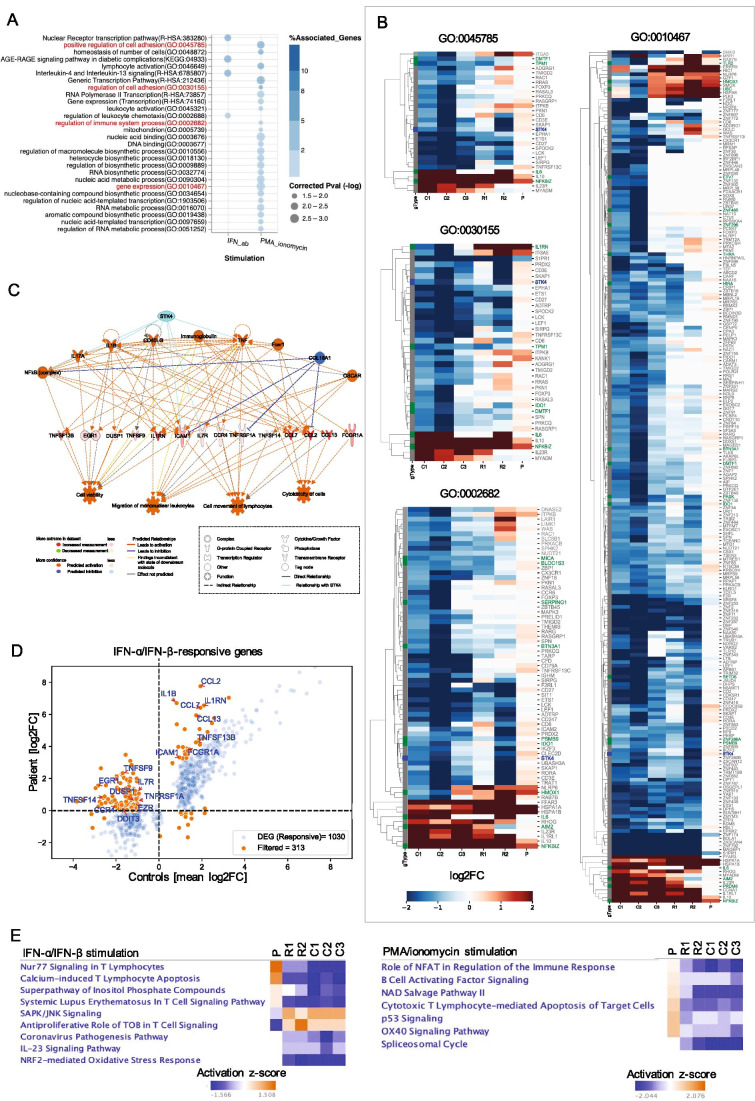
Gene enrichment analyses of IFN-a/IFN-b or PMA/ionomycin-responsive genes in PBMCs obtained from the STK4^−/−^ patient (P), his STK4^WT/−^ family members (R1, R2, and R3), and three unrelated STK4^WT/WT^ controls (C1, C2, and C3). Results were generated from one mRNA-Seq experiment. Each condition was assayed in duplicate. **A** Heatmap shows functionally grouped GO and KEGG/BioCarta pathway annotation networks (ClueGO) (*P* < 0.05 (BH correction); FDR < 0.05) that encompass either *STK4* (red font), or at least two genes from the STK4-interacting gene set (black font) (see Supplementary Figure S3C for a representation of STK4 interaction partners). Percentage of associated genes shown as a color gradient. Circle sizes represent the adjusted *P*-values for the gene enrichment analyses. **B** Heatmap shows the log_2_-transformed fold change values (log2FC) for genes that are part of selected gene networks shown in **A**. Upregulation and downregulation of genes is shown as a red and blue gradient, respectively. Genes that are part of the human interferome network [19] are labeled in green font; *STK4* is labeled in blue font. **C** Analysis of regulatory effects (IPA) of IFN-a/IFN-b-responsive genes that are dysregulated in the patient’s PBMCs (*P* < 0.05 (Fishers exact *t*-test); z-score > 2; consistency score 18.98). The top panel of nodes in the network graph depicts the predicted upstream regulators; the middle panel depicts the selected gene set, and the bottom panel depicts the best matching downstream effect. Solid cyan edges depict indirect relationships between nodes of the network and STK4 (Ingenuity Knowledge Base, Qiagen). **D** Scatter plot shows the log2FC values of the analyzed genes in the patient versus the mean FC values of the unrelated controls after stimulation with IFN-a/IFN-b. Orange symbols indicate responsive genes for which regulation was considered different in the patient compared to the controls (ratio < 0 or > 2). Genes that form part of the regulatory network in **C** are annotated. **E** Heatmap shows the results of a canonical pathway comparison analysis (IPA). The color gradient depicts z-score values. Pathways with *P* < 0.05 and z-score > 2 were considered significantly regulated. Red and blue indicate activated and repressed pathways, respectively. Only canonical pathways that were found to be differentially activated/repressed in the patient relative to the control subjects are shown

Finally, we performed mRNA-Seq of PBMCs isolated from the patient and stimulated or not with either IFN-a/IFN-b or PMA/ionomycin. Both parents and three unrelated, healthy controls were assessed for comparison. We filtered for genes responsive to in vitro stimulation among the unrelated control subjects and found a marked dysregulation, but not complete abrogation, of IFN-regulated gene expression in the patient (Supplementary Figure S3A and S3B). After an additional filtering step (see Methods, Online Resource 1), we performed gene enrichment analyses on the subsets of either IFN-a/IFN-b- or PMA/ionomycin-responsive DEGs that were dysregulated in the patient using ClueGO [17] and Cytoscape [18], thereby taking advantage of functionally grouped GO and KEGG/BioCarta pathway annotation networks. To gain a better mechanistic understanding of the molecular consequences of STK4 deficiency, we focused our analyses on gene networks that either encompass *STK4* or that include genes encoding products that interact directly with the STK4 protein (Supplementary Figure S3C). This analysis revealed several gene networks involved in the regulation of cell adhesion and leukocyte chemotaxis encompassing *STK4*, as well as gene networks involved in IFN-a/IFN-b- or mitogen-induced regulation of gene expression and biosynthesis processes that are typically associated with cell chemotaxis and adhesion-mediated cell signaling (Fig. 5A). Several of the genes that belong to these GO and pathway annotation networks are also previously reported type I interferon-responsive genes [19] (Fig. 5B). We also assessed regulatory effects on the subsets of IFN-a/IFN-b- or PMA/ionomycin-responsive DEGs using IPA and examined which genes or their upstream regulators are known to bind to, or are regulated by, STK4. This analysis revealed two regulatory networks of IFN-a/IFN-b-responsive genes encompassing several cytokine-, chemokine-, and adhesion factor- or receptor genes that are indirectly regulated by STK4, which was consistent with our ClueGO analysis. These genes are also involved in cytotoxicity and death of immune cells, as well as adhesion and migration of lymphocytes and mononuclear leukocytes (Fig. 5C, D, Supplementary Figure S3D-E). However, no regulatory effects networks were identified among PMA/ionomycin-responsive genes through the use of IPA (data not shown). Finally, we performed a canonical pathway comparison analysis of the entire sets of either IFN-a/IFN-b- or PMA/ionomycin-responsive genes using IPA to identify pathways that are differentially activated or inhibited in the patient’s cells compared to those of the controls. In response to IFNAR activation, nine pathways were differentially regulated in the patient compared to the controls, most of which are linked to T cell signaling and apoptosis, cell proliferation, oxidative stress, and, interestingly, IL-23 signaling (Fig. 5E). Similarly, we observed several pathways that are normally repressed following mitogen activation, but instead were highly activated or dysregulated in the patient’s cells. These included pathways primarily involved in T cell effector functions, T and B cell activation, cell cycle arrest, and apoptosis (Fig. 5E).

Given the apparent dysregulation of IL-23 signaling in the patient’s cells, we also examined absolute *IL-23* and *IFNG* gene expression, either at baseline or following stimulation with either PMA/ionomycin or IFN-a/IFN-b. In comparison to the control subjects, *IL-23* gene expression in the patient was highly impaired at baseline and largely unresponsive to PMA/ionomycin stimulation, whereas *IFNG* gene expression appeared normal (Supplementary Figure S1C and D).

## Discussion

In this study, we identified a novel stop-gain mutation in a patient with AR complete STK4 deficiency. This mutation was found to be located in a genomic region that encodes the coiled-coil domain of STK4, downstream of its protein kinase domain. We were unable to detect even a truncated STK4 protein in the patient using a monoclonal antibody to the N-terminal region of STK4, suggesting that protein expression of the mutated allele is completely abrogated due to nonsense-mediated decay.

In accordance with earlier case reports [5, 8, 20], we found that the PBMCs isolated from our patient had reduced fractions of CD4^+^ naïve, but increased effector memory, T helper cell subsets compared with those in the *STK4*^*wt/mut*^ family members and an unrelated *STK4*^*wt/wt*^ control. Furthermore, flow cytometric analysis showed a considerable proportion of the remaining T helper cell subset in the patient expressed higher levels of PD-1, and our RNA-Seq analyses revealed dysregulation of several pathways in the patient, suggesting elevated T cell exhaustion and impaired effector functions of the residual T cells. Whether this is a consequence of persistent EBV viremia [21–23] or an intrinsic feature of STK4 deficiency, or perhaps both, remains to be established. Previous studies have shown that EBV reactivation correlates with the expression of PD-1/PD-L1 antigens in patients with proliferative glomerulonephritis [24]. On the other hand, CD4^+^ T cell lymphopenia has also been reported in STK4-deficient patients in the absence of detectable EBV infection [4]. In addition, the patient presented with episodes of intermittent neutropenia, which is consistent with previous observations [2, 3, 6, 9, 13].

Our results also highlight that STK4 deficiency can lead to the impairment of a variety of T cell-independent and innate immune responses. Indeed, we detected a considerable proportion of CD56^bright^ NK cells in the PBMCs isolated from the patient. While these cells constitute only a small fraction of NK cells in the peripheral blood of healthy individuals, they represent the majority of NK cells in secondary lymphoid tissues. CD56^bright^ NK cells are thought to be NK cell precursors [25] and may have immunoregulatory properties [26]. We also observed a decreased fraction of pDCs in the patient’s peripheral blood. Whether this is an indirect consequence of active EBV infection, as shown in mouse studies [27], or whether low levels of pDCs contribute directly to a lack of EBV control, remains unclear. As reported by Jørgensen et al. [13], we also observed dysregulated type I and II IFN signaling in the patient’s cells. Interestingly, transcriptomic analysis of the patient’s PBMCs in response to IFNAR activation in vitro revealed a marked dysregulation of IFN-regulated gene expression, affecting interferon-stimulated genes (ISGs). Our enrichment analyses of either IFN-a/IFN-b- or PMA/ionomycin-responsive genes that showed differential expression between the patient and controls revealed several gene networks reminiscent of dysregulated cytokine-stimulated cell adhesion, leukocyte chemotaxis, and impaired T cell activation, likely resulting in T cell exhaustion and enhanced immune cell death. Dysregulation of these proinflammatory cytokines and chemokines has also been implicated in cancer pathogenesis [28]. Moreover, in accordance with our findings, Dang et al. demonstrated that leukocytes of patients with AR STK4 deficiency exhibited impaired chemotaxis after stimulation with CXCL11 and did not bind to ICAM-1 [5].

It cannot be ruled out that the dysregulated type I IFN-induced gene expression signature in the patient’s PBMCs is, in part, also a consequence of abnormal proportions of some leukocyte subsets. Indeed, we observed decreased fractions of pDCs, which are potent producers of type I IFN under in vitro and in vivo conditions [29]. Nonetheless, the short duration of the in vitro stimulation and gene expression experiments (2 h) and the low proportions of pDCs, CD56^bright^ NK cells, and effector memory T cell subsets relative to the total PBMC population make it less likely that abnormal proportions of some leukocytes subsets in the patient have a major effect on their PBMC transcriptome. Overall, the suboptimal IFN signaling may contribute to the T cell immunodeficiency and the vulnerability of STK4-deficient patients to viral infection and cancer development. However, overall fractions of CD19^+^ B cells (Fig. 2A) and IgG antibody responses to childhood vaccination (Supplementary Table S3) or common microbial infection (Fig. 3) did not appear to be diminished in our patient, apart from our observation that the antibodies were predominantly specific for HHV-4 and -5 antigens. Of note, a recent study in STK4^−/−^ mice and nine patients from five unrelated families with STK4 deficiency suggested that STK4 is required for normal humoral immunity since knockout mice and patients had reduced marginal zone B (MZB) cells as well as reduced numbers of innate-like B-1b cell subsets, while the overall fractions of circulating CD19^+^ B cells were normal, as in our patient [30]. This raises the possibility that patients with STK4 deficiency may also have a selective impairment in the ability to mount robust T cell-independent, polysaccharide-specific antibody responses to control natural infection with encapsulated bacteria, such as *H. influenzae*, *K. pneumoniae* and *S. pneumoniae*, which is consistent with the clinical history of our patient. Polysaccharide-specific antibody responses (or the lack of) are undetectable using the PhIP-Seq assay as it exclusively detects antibodies that target protein antigens and is limited in its capacity to detect conformational and post-translationally modified epitopes [31]. High efficacy of plain polysaccharide-based vaccines also depends on the maturation of MZB cells, which usually does not occur until the second year of life [32]. In our patient, the specific antibody responses were at the lower end of our laboratory reference range (Supplementary Table S3). However, anti-pneumococcal polysaccharide antibodies cannot be used as definitive markers of MZB cell-mediated immunity due to the introduction of the conjugate pneumococcal vaccine into the local routine immunization schedule. The literature shows variability in the specific antibody responses in STK4-deficient patients, ranging from normal to absent [30]. The history of infection with *H. influenzae*, *K. pneumonia*, and *S. pneumoniae* in our patient could have also interfered with the utility and interpretation of tests of responses to plain polysaccharide vaccines. Therefore, humoral immunity of patients with STK4 deficiency toward encapsulated bacteria requires further investigation.

We also demonstrated a profound impairment of *IL-23* gene expression in the patient’s PBMCs, both at baseline and following in vitro stimulation. IL-23 is produced by innate lymphoid cells, gamma-delta T cells, DCs, and macrophages, and it has been shown that *IL-23*-dependent IFN- immunity plays a pivotal role in controlling *Mycobacterium tuberculosis* (Mtb) infection [33]. It is therefore tempting to speculate that impaired *IL-23* gene expression contributed to patient’s susceptibility to pulmonary TB. Despite the clinical evidence of pulmonary TB, the patient’s QuantiFERON test result was indeterminate, which is likely to reflect a combination of cellular dysfunction and profound lymphopenia. Of note, Radwan et al. [7] also speculated that complications in a 9-year-old Egyptian boy with STK4 deficiency were associated with mycobacterial infection, although tuberculin skin-test results were negative, and the results from QuantiFERON tests were inconclusive.

It remains unclear whether malignancies in STK4-deficient patients are a secondary consequence of persistent EBV viremia, or whether such patients are inherently prone to malignancies due to dysregulation of oncogenes, even in the absence of EBV infection [4]. Interestingly, our RNA-Seq experiments revealed upregulation of mitogen-induced B cell-activating factor (BAFF) receptor gene (*TNFRSF13C*) expression in the patient, suggesting activation of BAFF signaling, in contrast to the controls where this pathway was inhibited following PMA/ionomycin stimulation (Fig. 5 and Supplementary Table S4). Studies in vitro and in mice have shown that EBV drives autonomous B cell proliferation [34], which also depends on T cell-independent survival signals provided by the BAFF receptor. Excessive BAFF levels have been implicated in several B-lineage malignancies [35–38], which have also been reported in the context of STK4 deficiency, with or without EBV viremia [2, 5–8, 10]. Our observations provide further mechanistic insights into the susceptibility of STK4-deficient patients to malignancies, although they do not allow firm conclusions to be drawn about the role of EBV in this process. Nonetheless, it is tempting to speculate that STK4-deficient patients, particularly those with persistent EBV viremia, may benefit from treatment with immune checkpoint inhibitors. Using a humanized mouse model, Ma et al. [39] demonstrated a direct beneficial effect of PD-1/CTLA-4 blockade mediated by monoclonal antibodies against PD-1 or CTLA-4 alone, or in combination, on EBV-associated B cell lymphomas, thereby providing further evidence in support of this hypothesis. However, TB reactivation or primary Mtb infections have also been reported in cancer patients who received checkpoint inhibitors [40–42]. Therefore, the potential therapeutic benefits of checkpoint inhibitors in patients with STK4 deficiency require further investigation.

## Methods

Detailed methods are provided in the Online Resources (Online Resource 1).

## Supplementary Information

Below is the link to the electronic supplementary material.

Supplementary file1 (PDF 1.86 MB)

Supplementary file2 (XLSX 27 KB)

## Data Availability

All processed data are available in the manuscript, the supplementary materials (Online Resources 1 and 2), and in a Bitbucket repository at https://bitbucket.org/taushifkhan/stk4_datacodes/src/master. Raw and processed sequence read data from the PhIP-Seq experiments are available from the corresponding author on reasonable request. Raw and processed RNA-Seq data are available at the Gene Expression Omnibus (GEO), series accession number GSE166761.

## Code Availability

Python in-house scripts used in this study are available at https://bitbucket.org/taushifkhan/stk4_datacodes/src/master.
