# Supplementary material for: A Novel STK4 Mutation Impairs T Cell Immunity Through Dysregulation of Cytokine-Induced Adhesion and Chemotaxis Genes
Source: J Clin Immunol. 2021 Aug 24;41(8):1839–52. doi: 10.1007/s10875-021-01115-2 (PMC8604862; doi:10.1007/s10875-021-01115-2)
Supplement: Supplementary file 1 — Supplementary file1 (PDF 1.86 MB) [file 10875_2021_1115_MOESM1_ESM.pdf]

Online Resource 1

Supplementary Material for

A novel STK4 mutation impairs T cell immunity through dysregulation of cytokine-induced adhesion and chemotaxis genes.

Andrea Guennoun, Salim Bougarn, Taushif Khan, Rafah Mackeh, Mahbuba Rahman, Fatima Al-Ali, Manar Ata, Waleed Aamer, Debra Prosser, Tanwir Habib, Evonne Chin-Smith, Khawla Al-Darwish, Qian Zhang, Alya Al-Shakaki, Amal Robay, Ronald G. Crystal, Khalid Fakhro, Amal Al-Naimi, Eman Al Maslamani, Amjad Tuffaha, Ibrahim Janahi, Mohammad Janahi, Donald R. Love, Mohammed Y. Karim, Bernice Lo, Amel Hassan, Mehdi Adeli, Nico Marr

*Address correspondence to: Nico Marr, Sidra Medicine, Research Branch, Doha, Qatar, PO BOX 26999, E-mail: nmarr@sidra.org

This PDF file includes:

- Method details
- Supplementary Tables S1 to S4
- Supplementary Figures S1 to S3

Methods

Whole genome sequencing. Genomic DNA (gDNA) was isolated from the peripheral blood of the subjects using DNeasy® Blood & Tissue Kits (Qiagen, Germantown, MD, USA). Whole genome sequences were obtained using a standard library preparation protocol and a HiSeq-X platform (Illumina, San Diego, CA, USA) to generate 150-bp paired-end sequences. The raw sequencing reads were mapped to the reference genome (GRCh37, hg19) using BWA (version 0.7.15) (43), and genetic variants were called with the HaplotypeCaller in the GATK suite (v.4.0) (44). The Variant Call Format (VCF) file, was annotated using SnpEff v.4.3 (45) and filtered for the candidate fitting the following criteria: (a) In the coding region including the exonic, splice-site region, (b) rare (<1%) in all mutation databases (i.e., 1,000 genomes, gnomAD, ExAC, and ESP6500), and (c) co-segregates with the phenotype in the family and follows a specific mode of inheritance (e.g., AR). The final list of variants was prioritized based on the literature search and whether any of the associated genes had been linked to the patient's symptoms, such as known Mendelian genes (https://omim.org).

Sanger sequencing. Primers seq_STK4_Ex8F (5'-GTCCGAAGCACAAAGAGAAAGA-3') and seq_STK4_Ex8R (5'-CCAGCTCCAAGTTGATCCAATA-3') were designed to flank exon 8 of the STK4 gene (NM_006282.5; LRG_535t1), checked for underlying single nucleotide polymorphisms, and synthesized as described previously (46). The forward and reverse primers were tailed with M13 sequences: CACGACGTTGTAAAACGAC (added to the 5' end of the forward primer), and CAGGAAACAGCTATGACC (added to the 5' end of the reverse primer). DNA was extracted from peripheral blood using a QIAsymphony® SP and QIAsymphony DSP DNA Midi Kit (Qiagen). DNA quantity and quality were assessed using a NanoDrop® ND-1000 spectrophotometer (Thermo Scientific, Wilmington, DE, USA). gDNA (50 ng) was subjected to conventional PCR using the following conditions: 95°C for 10 min, 34 cycles of 95°C for 30 s, 60°C for 30 s and 72°C for 45 s, and incubation at 72°C for 5 min. Amplicons were checked for size and non-specific amplification prior to ExoSAP-IT cleanup, cycle sequencing using primers comprising the M13 tails, and BigDyeXTerminator cleanup followed by loading onto an Applied Biosystems model 3500XL Genetic Analyzer (Thermo Fisher Scientific, Waltham, MA, USA). Sequence data were analyzed using Applied Biosystems Sequence Analysis software, and Mutation Surveyor software.

RT-qPCR analysis of STK4 gene expression levels. PBMCs were isolated from the patient, parents and healthy donors by Ficoll-gradient centrifugation. Cells were then incubated with T cell activation and expansion beads (anti-CD2/CD3/CD28; Miltenyi Biotec, Gaithersburg, MD, USA) for 10 days in 5% CO2 at 37°C. On day 3, the medium was supplemented with IL-2 (100 U/ml) to promote cell proliferation. RNA was extracted from cells (10^6) using TRIzol lysis reagent (Thermo Fisher Scientific, Carlsbad, CA, USA) according to the manufacturer's instructions. Briefly, cells (10^6) were lysed in 1 ml TRIzol for 3 min. Chloroform (140 μl) was then added and the phases were separated by centrifugation (16,000 xg at 4°C) RNA-containing liquid phase was mixed with an equal volume of isopropanol and incubated for 10 min at room temperature for RNA precipitation. After centrifugation, precipitated RNAs were de-salted by incubation with 1/10 volume of 5 M NH4OAc and 2.5 volumes of 100% cold ethanol, followed by washing with 75% ethanol and final elution in nuclease free water. The *STK4* transcript was quantified using Fast SYBR Green Master Mix (Applied Biosciences) with the following primers: forward 5'-TGGAGACGGTACAGCTGAGG-3'; reverse 5'-ATAGCAACAATCTGGCCGGT-3'. All reactions were performed in triplicate using a QuantStudio 6K Flex real-time PCR System (Applied Biosciences). Data were normalized to the expression of the housekeeping gene *RPLP0* (ribosomal protein, large, P0) and mean ΔCt^{-1} values were plotted.

Western blot analysis. T cell lysates containing 20 μg total protein were separated by electrophoresis (4%–15% tris-glycine Bis-Tris Gels; BioRad, Hercules, CA, USA) and transferred to a polyvinylidene fluoride membrane. After blocking with 5% non-fat milk in tris-buffered saline (TBS) for 1 h, membranes were incubated overnight at 4°C in TBS in the presence of anti-recombinant STK4 antibody (EP1465Y, 1:5,000, Abcam), α-tubulin monoclonal antibody (diluted 1:10,000) (Cell Signaling Technology, Denvers, MA, USA, catalog no. 3873), or anti-β-actin antibody (diluted 1:5,000) (Santa Cruz Biotechnology, Dallas, TX, USA, catalog no. sc-47778). Membranes were then washed (3 x 5 min) with TBS with 0.1% Tween-20 (TBS-T) and then incubated in TBS with a 1:5,000 dilution of either horse-radish peroxidase (HRP)-conjugated goat anti-rabbit (Invitrogen, Frederick, MD, USA, catalog no. 65-6120), or goat anti-mouse (Invitrogen, catalog no. 31430) secondary antibody, for 1 h at room temperature. Membranes were washed again (3 x 5 min) in TBS-T. Immunoreactive proteins were visualized using enhanced chemiluminescence (ECL™) (Amersham BioSciences UK Ltd., Buckinghamshire, UK) according to the manufacturer's instructions. Images were acquired using the ChemiDoc MP imaging system (BioRad).

In vitro stimulation of whole blood and high-throughput gene expression analysis. Peripheral whole blood (WB) was collected into sodium heparin vacutainer tubes. After incubation for 2 h at room temperature, 2 ml WB was diluted with an equal amount of RPMI 1640 media containing GlutaMax™ (Gibco). Samples were then stimulated for 2 h at 37°C with the following agonists (final concentrations are shown): ultrapure LPS of *E. coli* K12 (InvivoGen, San Diego, CA, USA) at 10 ng/ml; R848 (InvivoGen) at 3 µg/ml; Poly(I:C) (high molecular weight) (InvivoGen) at 25 µg/ml; recombinant human IL-1β (R&D Systems Inc., Minneapolis, MN, USA) at 20 ng/ml; a 1:1 mix of recombinant human IFN-α2 and IFN-β (R&D Systems) at 1,000 IU/ml each; recombinant human IFN-γ (R&D Systems) at 1,000 IU/ml; recombinant human TNF-α (R&D Systems) at 20 ng/ml; 5'ppp-dsRNA/LyoVec™ (InvivoGen) at 1 µg/ml; Poly(dA:dT)/LyoVec™ (InvivoGen) at 10 ng/ml; 3'3'-cGAMP/LyoVec™ (InvivoGen) at 10 µg/ml; MDP (InvivoGen) at 10 ng/ml; Dynabeads™ Human T-Activator CD3/CD28 (Invitrogen) at 400,000 beads/ml; AffiniPure $F(ab')_2$ fragment goat anti-human IgG + IgM (H+L) (Jackson ImmunoResearch) at 10 µg/ml; or PMA/ionomycin calcium salt (Sigma) at 40 ng/ml and 1µg/ml. Similarly, cells were incubated for 2 h at 37°C with LyoVec™ alone or RPMI medium with and without LyoVec™ (unstimulated control samples). Subsequently, Tempus™ solution (3 volumes, Applied Biosystems) was added for cell lysis and RNA stabilization before total RNA was isolated using the Tempus™ Spin RNA isolation kit (Applied Biosystems). RNA quality and quantity were assessed using an Agilent 2100 Bioanalyzer (Agilent Technologies, Santa Clara, CA, USA) and NanoDrop 1000 (Thermo Fisher Scientific). The cDNAs of all 180 target genes and six housekeeping genes were generated from 40 ng total RNA using a reverse transcription master mix (Fluidigm) and quantified using custom Delta Gene™ assays in combination with the 96.96 Dynamic Array™ IFCs and BioMark™ HD microfluidic system (Fluidigm) in accordance with the manufacturer's instructions. All 180 target genes and six housekeeping genes (**Supplementary Table S5**) were selected *a priori* from a larger set of 1,088 transcripts, represented by 66 sets of co-expressed genes (transcriptional modules), which had previously been found to be responsive in WB stimulation with purified PRR agonists, recombinant cytokines and pyogenic bacteria (16). For a total of 60 modules, we selected three representative genes per transcriptional module based on their closeness to the median absolute expression of all transcripts assigned to a given module. Six modules were excluded from the analysis due to limited functional annotation. Housekeeping genes were selected from the same dataset (GSE25742) as previously described (47). Expression levels of the 180 target genes were quantified relative to the six housekeeping genes using the $2^{-\Delta\Delta Ct}$ method (48). We evaluated fold changes (FC) in expression as $\log_2$-transformed expression level

(log2FC) for each target gene under each stimulation condition and filtered for genes with a |log2FC| ≥1 in at least two samples. We computed z-score values of the log2FC for each gene and condition in the patient using two unrelated control subjects and the three heterozygous family members as controls. Finally, we used principal component analysis (PCA) to estimate the variance in z-scores across samples. The first two principal components described approximately 75% of the variance in the z-score profile of samples and a distinct segregation of patient and control subjects was observed (**Supplementary Figure S2**). We extracted the contribution factor for each gene from the PCA and filtered for pairs of target gene and stimulation condition that showed a variance of |log2FC| >1 between the patient and control subjects, resulting in the identification of 28 target genes that were differentially expressed under one or more (i.e., up to eight) *in vitro* stimulation conditions (**Figure 4 and Supplementary Table S6**).

PBMC stimulation and RNA-Seq analysis. PBMCs were isolated from peripheral blood by Ficoll-Paque (GE Healthcare) density gradient centrifugation according to the manufacturer's instructions and frozen in fetal bovine serum containing 20% dimethyl sulfoxide. For *in vitro* stimulation, PBMCs where thawed, rested for 24 h and then stimulated with a 1:1 mix of recombinant human IFN-α2 and IFN-β (R&D Systems), PMA/ionomycin, or left unstimulated as described above. Total RNA was isolated using the RNeasy Mini Kit (Qiagen) according to the manufacturer's instructions. RNA integrity and purity were evaluated as described above. cDNA was generated using the SMARTer v4 Ultra® Low Input RNA for Sequencing Kit (Takara Bio). Resulting cDNA was quantified and size-controlled using a Bioanalyzer 2100 (Agilent Technologies Genomics). cDNA was normalized to 1 ng/μl and libraries were prepared using the Nextera XT DNA Library Preparation Kit (Illumina) and the Nextera XT Index Kit v2 set A (Illumina), respectively, in accordance with the manufacturer's instructions. After 150-bp paired-end sequencing to approximately 20 million reads per sample using a HiSeq4000 system (Illumina), raw reads were aligned to the UCSC human genome assembly version hg38 using STAR aligner (49). The HTSeq-count (50) tool was used to count the number of reads that mapped to each gene feature. Read counts were adjusted to mitigate batch effects by negative binomial regression models using comBat-Seq (51). After read counts were normalized, we removed genes expressed at low levels using the HTSFilter and the default protocol. We calculated log2FC values for each subject and stimulation condition relative to the unstimulated condition of the same subject using edgeR (52). Genes with a |log2FC| ≥1 in at least two out of three unrelated controls were considered *in vitro* stimulation-responsive genes (**Supplementary Figure S3A**). Residual responses were calculated as described previously (53). We focused our downstream analysis on the responsive genes that differed in their expression in the patient's cells by calculating the ratio of the mean FC values in the controls and that of the patient (**Supplementary Figure S3B**). We then performed gene enrichment analyses and biological interpretation in the subset of responsive genes with a ratio <0 or ≥2 using ClueGO (v2.5.7) (17) and Cytoscape (v 3.7.1) (18) with an updated Gene Ontology (GO)/Kyoto Encyclopedia of Genes and Genomes (KEGG) release (May 8, 2021). The identified gene networks (*P* and FDR ≤0.05) were further grouped to distinguish those that encompass STK4, those that encompass genes encoding products shown to interact directly with STK4, and those that appeared to be indirectly affected in the STK4-deficient patient. The STK4-interacting gene set used for this analysis was extracted from the human reference interactome mapping project (54) and functional protein-protein interactions were verified using the STRING database (v. 11.0b) (55) (**Supplementary Figure S3C**). We also examined the expression of selected responsive genes that were part of the GO and pathway annotation networks identified in our study and that also belong to the human interferome, an expanded network of type I interferon-responsive genes described earlier (19). Statistical data analysis was performed and visualized using in-house python (v. 3.7) scripts (https://bitbucket.org/taushifkhan/stk4_datacodes/src/master/). Additionally, we analyzed

regulator effects in the subset of responsive genes and performed a canonical pathway comparison analysis ($P \leq 0.05$; z-score ≥ 2) through the use of the Ingenuity Pathway Analysis (IPA) application (Qiagen, https://www.qiagenbioinformatics.com/products/ingenuity-pathway-analysis) (56). For the analysis of regulatory effects, we considered only experimentally observed relationships between upstream regulators and responsive genes in our dataset.

Phage immunoprecipitation-sequencing (PhIP-Seq). The VirScan phage library used for PhIP-Seq was obtained from S. Elledge (Brigham and Women's Hospital and Harvard Medical School, Boston, MA, USA). PhIP-Seq was performed as described previously (31, 57, 58) using an expanded version of the original VirScan phage library (59). We computed species-specific adjusted score values as described earlier (58, 60). Pooled human plasma used for IVIg (Privigen® CSL Behring AG) and human IgG-depleted serum (Molecular Innovations) served as controls.

Flow cytometry. Lymphocyte subsets were assessed using Duraclone assays (Beckman Coulter Life Sciences, Indianapolis, Indiana, USA) to delineate major lymphocyte subsets (IM Phenotyping Basic) and more specifically, T cell and dendritic cell subsets (IM T cell and IM DCs) according to the manufacturer's protocol. Samples were analyzed with a FACSymphony A5 Flow Cytometer (BD Biosciences) and FlowJo 10.5.2 software.

Absolute blood cell counts, total immunoglobulin levels, vaccine-specific antibody measurements and QuantiFERON-TB Gold ELISA. Complete blood cell and lymphocyte counts, total immunoglobulin measurements (i.e., IgG, IgA, IgM), vaccine-specific antibody measurements (i.e., anti-diphtheria toxoid IgG, anti-*Haemophilus influenzae* Type B, polyclonal anti-pneumococcus capsular antibody, anti-tetanus toxoid antibody) and whole blood IFN-γ measurements in response to Mtb peptide antigens (QuantiFERON-TB Gold ELISA, Qiagen) were carried out and interpreted by laboratories accredited by the College of American Pathologists (CAP) following the manufacturer's instructions and clinical guidelines. Normal ranges are provided in tables and figures as appropriate. An "indeterminate" test result for the QuantiFERON-TB Gold ELISA indicated the patient's lymphocytes also did not respond to mitogen stimulation (as seen in immunodeficiency, lymphopenia, overwhelming infection, malnutrition, and with immunosuppressive medication).

Statistical analysis. For PhIP-Seq experiments, we imputed $-\log_{10}(P\text{-values})$ by fitting a zero-inflated generalized Poisson model to the distribution of output counts followed by regression of the parameters for each peptide sequence based on the input read count. Peptides that passed a reproducibility threshold of $-\log_{10}(P\text{-value}) \geq 2.3$ in two technical sample replicates were considered significantly enriched. For gene enrichment analyses, we generated functionally grouped gene ontology and KEGG/BioCarta pathway annotation networks (ClueGO) as described above. A P value <0.05 [BH correction] and FDR <0.05 was considered significant. For canonical pathway comparison analyses using IPA, pathways with a P value <0.05 (Fisher's exact t-test) and |z-score| >2 were considered activated or repressed, as detailed in the figure legends.

Supplementary Tables

Supplementary Table S1. Epstein–Barr virus (EBV) load in the patient's serum.

Sample collection no	Age (years)	EBV viral load (IU/ml)
1	12	1205
2	12	1573
3	12	100
4	12	925
5	14	1571

Supplementary Table S2. Neutrophil and lymphocyte counts for the patient at 11 to 15 years of age.

Sample collection no	Age (years)	Neutrophils: Neutrophil counts of P ($x10^9$)	Neutrophils: Reference range	Lymphocytes: Lymphocyte counts of P ($x10^9$)	Lymphocytes: Reference range
1	11	2.36	1.63–7.55	0.41 [c]	0.97–3.96
2	11	2.32	1.63–7.55	0.31 [c]	0.97–3.96
3	12[a]	6.8	2–7	0.39 [c]	1.5–6.5
4	12	2.6	2–7	0.6 [c]	1.5–6.5
5	12	1.5 [c]	2–7	0.5 [c]	1.5–6.5
6	12	1.7 [c]	2–7	0.5 [c]	1.5–6.5
7	12	2.6	2–7	0.2 [c]	1.5–6.5
8	12	1.7 [c]	2–7	0.4 [c]	1.5–6.5
9	12	1.5 [c]	2–7	0.4 [c]	1.5–6.5
10	12	1.7 [c]	2–7	0.4 [c]	1.5–6.5
11	12	1.6 [c]	2–7	0.4 [c]	1.5–6.5
12	12	3.1	2–7	0.2 [c]	1.5–6.5
13	13[b]	7.4	2–7	0.4 [c]	1.5–6.5
14	13	2.8	2–7	0.2 [c]	1.5–6.5
15	13	2.4	2–7	0.2 [c]	1.5–6.5
16	13	3.2	2–7	0.2 [c]	1.5–6.5
17	13	3.3	2–7	0.3 [c]	1.5–6.5
18	13	2.3	2–7	0.3 [c]	1.5–6.5
19	13	3.5	2–7	0.2 [c]	1.5–6.5
20	13	2.7	2–7	0.3 [c]	1.5–6.5
21	13	2	2–7	0.5 [c]	1.5–6.5
22	15	1.7 [c]	2–8	0.3 [c]	0.7-3.5

[a] The sample was obtained during a clinical episode of an acute respiratory infection during which a sputum culture revealed abundant growth of *Haemophilus influenzae*, modest growth of methicillin-resistant *Staphylococcus aureus*, heavy growth of *Streptococcus pneumoniae* and light growth of oropharyngeal flora.
[b] The sample was obtained while the patient was hospitalized for suspected TB reactivation. Bronchoalveolar lavage analysis showed heavy growth of *H. influenzae* and light growth of *Klebsiella pneumoniae* with extended spectrum beta-lactamase activity (resistant to all penicillins, cephalosporins and aztreonam); however, the sample was TB negative.
[c] Results outside the normal, age-adjusted range are labeled in blue (low levels).

Supplementary Table S3. Clinical laboratory test results for the patient from four different clinical visits between 10 and 13 years of age.

Clinical test	Clinical visit no.				Reference range[b]
	1	2	3	4	
IgG (mg/dL)				1467	59–1600
		1400	1800 [a]		700–1600
IgA (mg/dL)				475 [a]	29–251
		456 [a]	554 [a]		70–400
IgM (mg/dL)				25 [a]	26–184
		29.7 [a]	29.2 [a]		40–230
IgE (units/L)			303 [a]	163 [a]	0–114
IgG Sub 1 (mg/dL)		1080 [a]		1160 [a]	362–1027
IgG Sub 2 (mg/dL)		154		133	81–472
IgG Sub 3 (mg/dL)		7.2 [a]		10.4 [a]	13.8–105.8
IgG Sub 4 (mg/dL)		31.9		13.8	4.9–198.5
Anti-diphtheria toxoid IgG (IU/ml)	0.032	0.067		0.148	Positive >0.01
Anti-haemophilus Flu Type B (mg/L)	0.19	0.18		0.11 [a]	Positive >0.15
Anti-pneumococcus capsular poly Ab (mg/L)	4.07	11.7		10.97	Positive >3.3
Anti-tetanus toxoid antibody (IU/ml)	0.04	0.09		0.52	Positive >0.01
QuantiFERON Tb	Indet.[c]				

[a] Results outside the normal, age-adjusted range are color coded in red (high) and blue (low).
[b] Two reference ranges for IgG, IgA and IgM are indicated, which are adjusted according to the patient's respective age.
[c] Indeterminate QuantiFERON test result, i.e., the patient's lymphocytes did not respond to mitogen stimulation.

Supplementary Table S4. Dysregulated genes identified by gene enrichment analysis using QIAGEN Ingenuity Pathway Analysis. The listed genes are implicated in the canonical pathways shown in Figure 5E.

Gene	Stimulation	Number and names of pathways	Expression level in patient	Expression levels in controls and parents
TRGV9	IFN-α/IFN-β	5[a,c,f,g,i]	up	down
CD3D	IFN-α/IFN-β	4[a,c,f,i]	up	down
CD3G	IFN-α/IFN-β	4[a,c,f,i]	up	down
MAP2K3	IFN-α/IFN-β	2[e,i]	up	down
GADD45A	IFN-α/IFN-β	2[g,i]	up	down
DDIT3	IFN-α/IFN-β	1[b]	up	down
IL23A	IFN-α/IFN-β	1[d]	down	up
CLPP	IFN-α/IFN-β	1[e]	up	down
PTPRO	IFN-α/IFN-β	1[h]	down	up
CD3E	PMA/ionomycin	3[m,o,j]	up	down
CD247	PMA/ionomycin	3[m,o,j]	up	down
NMRK1	PMA/ionomycin	1[l]	up	down
NT5C3B	PMA/ionomycin	1[l]	up	down
TNFRSF13C	PMA/ionomycin	1[k]	up	down
PRKCQ	PMA/ionomycin	1[j]	up	down
GNB5	PMA/ionomycin	1[j]	up	down
RCAN3	PMA/ionomycin	1	up	down
LCK	PMA/ionomycin	1[j]	up	down
DDX39B	PMA/ionomycin	1[p]	up	down
SF3A3	PMA/ionomycin	1[p]	up	down
PIDD1	PMA/ionomycin	1[n]	up	down

[a] Antiproliferative role of TOB in T cell signaling; [b] Coronavirus pathogenesis pathway; [c] Calcium-induced T lymphocyte apoptosis; [d] IL-23 signaling pathway; [e] NRF2-mediated oxidative stress response; [f] Nur77 signaling in T lymphocytes; [g] SAPK/JNK signaling; [h] Superpathway of inositol phosphate compounds; [i] Systemic lupus erythematosus in T cell signaling pathway; [j] Role of NFAT in regulation of the immune response; [k] B cell activating factor signaling; [l] NAD salvage pathway II; [m] Cytotoxic T lymphocyte-mediated apoptosis of target cells; [n] p53 signaling; [o] OX40 signaling pathway; [p] Spliceosomal cycle.

Supplementary Figures

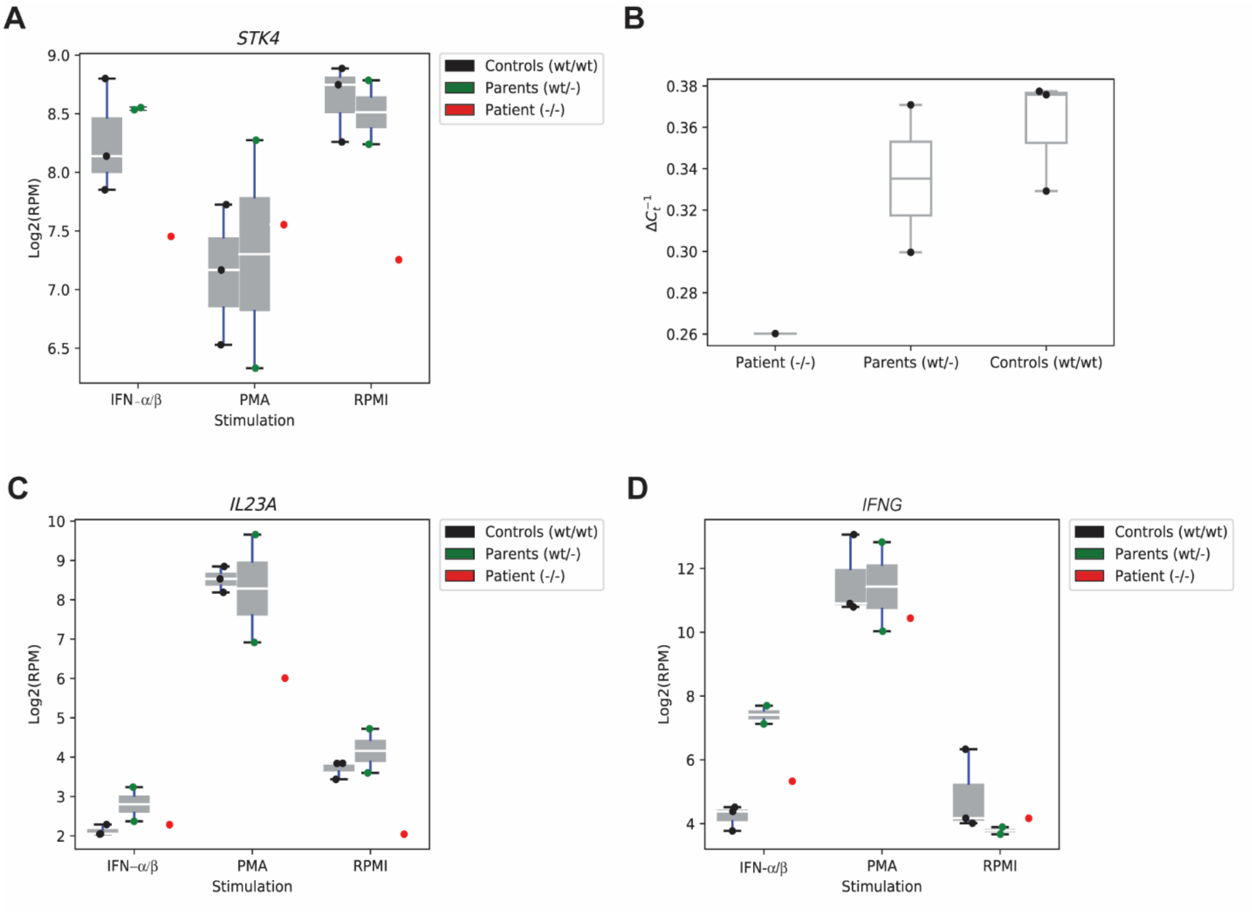

Supplementary Figure S1. Transcript levels of *STK4*, *IL23* and *IFNG* genes. A, Box plots of the normalized read counts for the *STK4* gene from the RNA-Seq analysis of PBMCs from the STK4$^{-/-}$ patient, the patient's STK4$^{wt/-}$ parents and three unrelated, healthy STK4$^{wt/wt}$ controls; PBMCs were stimulated with PMA/ionomycin, IFN-α/IFN-β, or left unstimulated (RPMI). For each condition, stimulations were performed in duplicate and the normalized read counts of the same individual and condition were averaged. **B,** Quantitative reverse transcriptase PCR analysis of the *STK4* transcript in PBMC-derived T cells from the STK4$^{-/-}$ patient, the patient's STK4$^{wt/-}$ parents and three unrelated, healthy STK4$^{wt/wt}$ controls. Data are normalized to the expression of the housekeeping gene RPLP0 (ribosomal protein, large, P0) and mean ΔCt $^{-1}$ values are plotted. Data are representative of one experiment, performed in triplicate. **C and D,** Box plots of the normalized read counts for the **(C)** *IL23* and **(D)** *IFNG* genes from the RNA-Seq analysis of PBMCs from the STK4$^{-/-}$ patient, the patient's STK4$^{wt/-}$ parents and three unrelated, healthy STK4$^{wt/wt}$ controls; PBMCs were stimulated with PMA/ionomycin, IFN-α/IFN-β, or left unstimulated (RPMI). For each condition, stimulations were performed in duplicate and the normalized read counts of the same individual and condition were averaged. RPM, reads per million.

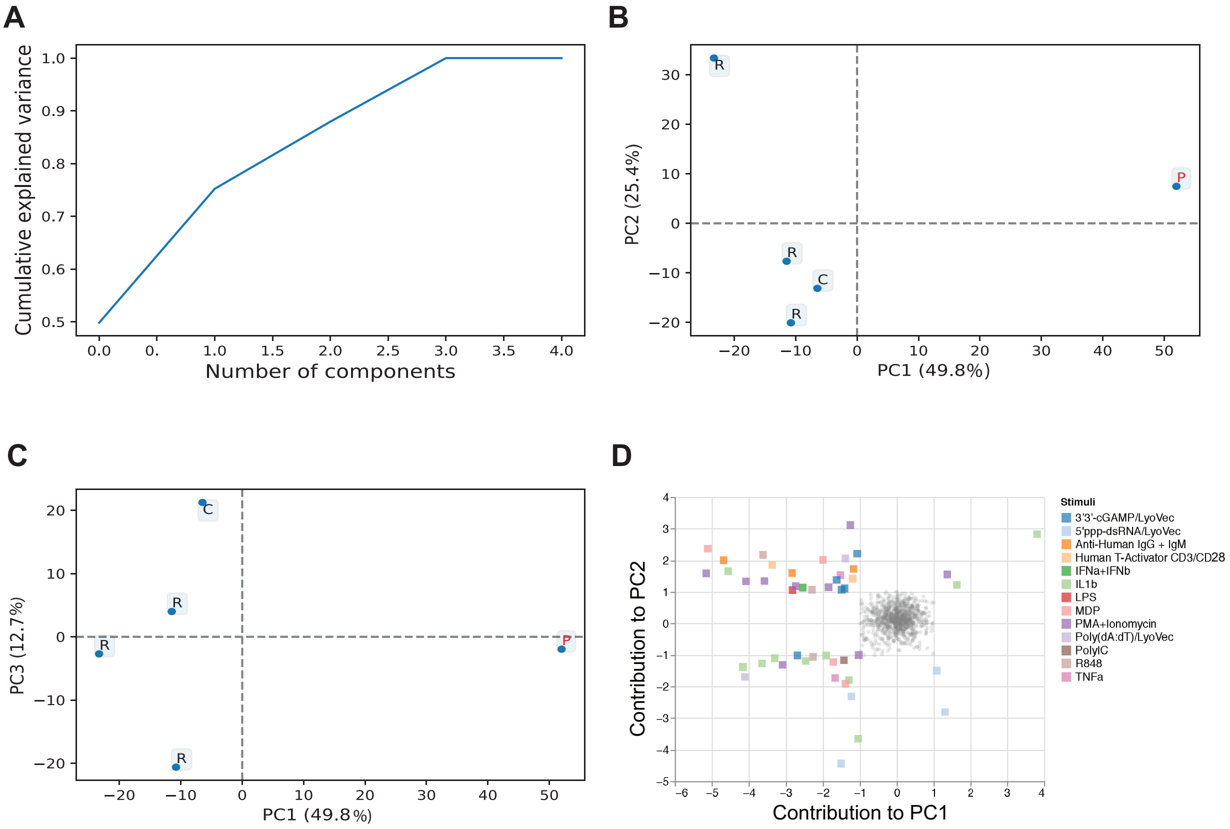

Supplementary Figure S2. Principal component analysis (PCA) of the 180 target genes from 14 stimulation conditions in the patient (P) and controls, including his STK4$^{wt/-}$ relatives (R) and an unrelated STK4$^{wt/wt}$ control (C). (**A**) Cumulative sum of explained variance over the number of components. (**B and C**) Sample projection in principal components 1 and 2**,** or 1 and 3. (**D**) Contribution of different genes to the first two principal components. Data points (gene-stimulation pairs) with a variance of |log2FC| ≥1 are color-coded according to the stimulation condition shown in the legend.

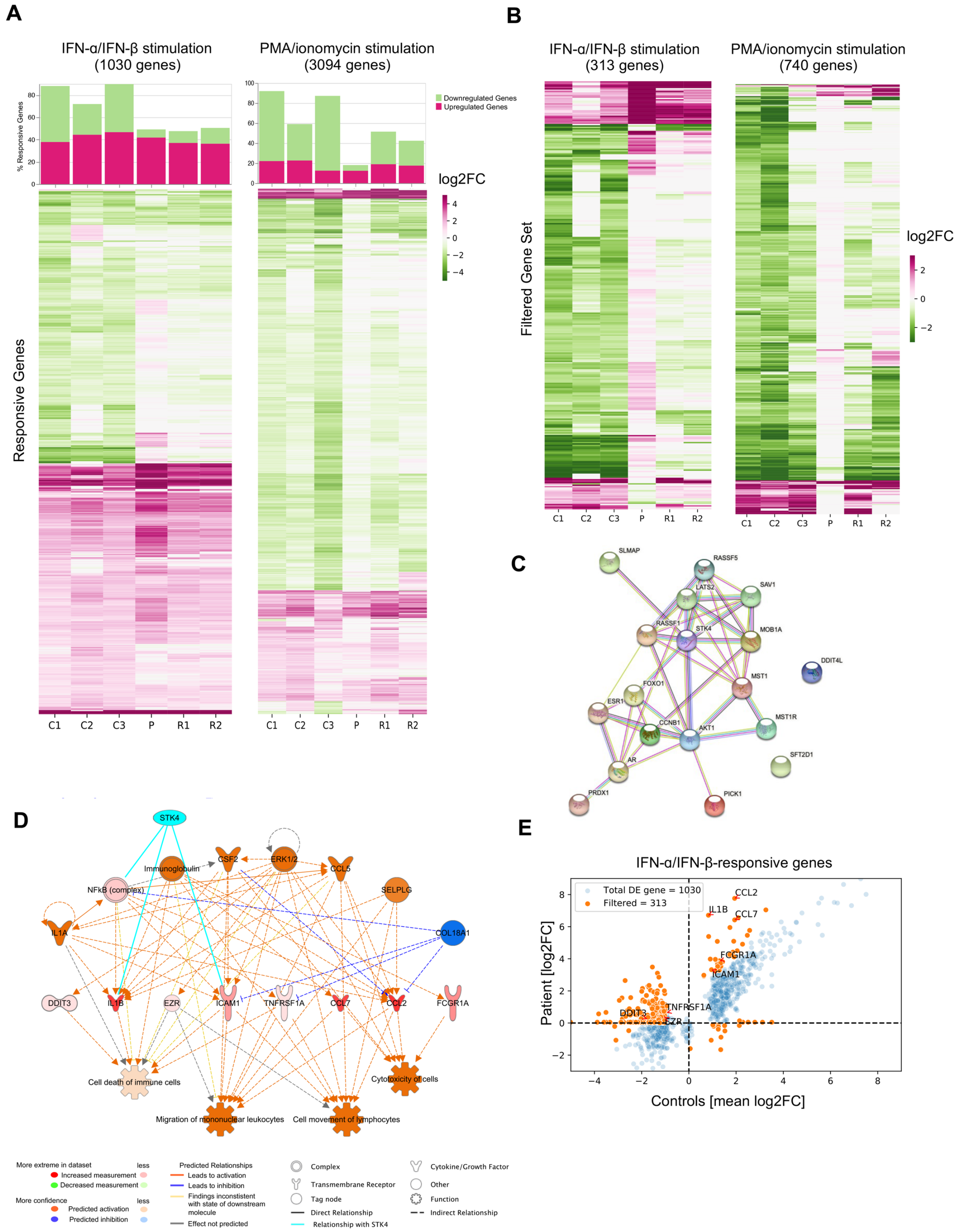

Supplementary Figure S3. RNA-Seq and gene enrichment analyses of stimulated PBMCs. Gene expression profiling of the STK4$^{-/-}$ patient (P), his STK4$^{WT/-}$ family members (R1, R2 and R3) as well as two unrelated STK4$^{WT/WT}$ controls (C1 and C2). Results were obtained from one

experiment, performed in duplicate. (**A**) Heatmaps show log2-transformed fold change values (log2FC) of genes responsive to *in vitro* stimulation with either recombinant human IFN-α/IFN-β (left), or PMA/ionomycin (right) in at least two out of three unrelated controls (|log2FC| >1). Pink and green indicate upregulated and downregulated genes, respectively. (**B**) Heatmap shows a subset of genes in (**A**) that differed in their expression in the patient's cells relative to the unrelated control subjects. (**C**) Protein-protein interaction network including STK4 (STRING). (**D**) Analysis of regulatory effects (IPA) of IFN-α/IFN-β–responsive genes that are dysregulated in the patient's PBMCs (P < 0.05 [Fishers exact *t*-test]; z-score >2; score consistency score: 19.8). The top panel of nodes in the network graph depicts the predicted upstream regulators; the middle panel depicts the selected gene set, and the bottom panel depicts the best matching downstream effect. Solid cyan edges depict indirect relationships between nodes of the network and STK4 (Ingenuity Knowledge Base, Qiagen). (**E**) Scatter plot shows the log2FC values of the analyzed genes in the patient versus the mean FC values of the unrelated controls after stimulation with IFN-α/IFN-β. Orange symbols indicate responsive genes for which regulation was considered different in the patient compared to the controls (ratio <0 or >2). Genes that form part of the regulatory network in (**D**) are annotated.
